# Gut Microbiota Dysbiosis and Neuroinflammation in Alzheimer’s Disease: a Systematic Review of Mechanistic Insights

**DOI:** 10.1007/s12035-026-05914-9

**Published:** 2026-05-12

**Authors:** Sofia Katsigianni, Effrosyni Koutsouraki

**Affiliations:** School of Medicine, Aristotle University, Thessaloniki, Greece

**Keywords:** Alzheimer’s disease, Cognitive decline, Gut-brain axis, Gut microbiota, Inflammatory signaling, Neuroinflammation, SCFA

## Abstract

This systematic review aims to identify microbial species associated with Alzheimer’s disease and clarify how they influence Alzheimer’s disease pathology, including immune system interactions and blood–brain barrier integrity. Following Preferred Reporting Items for Systematic Reviews and Meta-Analyses guidelines, 18 studies were analyzed, revealing significant differences in gut microbiota composition between patients with Alzheimer’s disease and controls. Key findings include reduced beneficial bacteria, such as *Faecalibacterium prausnitzii* and *Roseburia hominis* and increased pro-inflammatory species like *Escherichia coli*. Mechanistically, *Escherichia coli* produces lipopolysaccharides, triggering the release of pro-inflammatory cytokines such as tumor necrosis factor-alpha. This response correlates with elevated YKL-40 levels and reduced beneficial bacteria like *Turicibacter*. Additionally, reductions in butyrate-producing bacteria impair blood–brain barrier integrity, while bile acid metabolism disruptions impair signaling pathways, exacerbating amyloid-beta aggregation. These findings highlight the interplay between gut microbiota-derived metabolites and neuroinflammation, suggesting that targeting the gut microbiota for novel diagnostic and therapeutic approaches in Alzheimer’s disease.

## Introduction

Alzheimer’s disease (AD) is a progressive neurodegenerative disorder characterized by cognitive decline, memory impairment and increasing functional dependence [1]. Clinically, AD exists along a continuum, beginning with subjective cognitive decline (SCD), progressing to mild cognitive impairment (MCI) and ultimately leading to dementia [1, 2]. Pathologically, AD is defined by extracellular amyloid plaques, intracellular neurofibrillary tangles of hyperphosphorylated tau, and progressive neuronal loss [2].

Although the precise etiology of AD remains unclear, growing evidence underscores the gut-brain axis as a contributor to disease pathogenesis [3]. The gut microbiota (GM) plays a central role in host metabolism, immune regulation, and neuroimmune communication, and its disruption has been associated with neurodegenerative processes [4–7]. Alterations in microbial composition have been linked to immune dysregulation, chronic inflammation, and pathways relevant to amyloid pathology and neuronal function [8–12]. Multi-omics approaches further suggest that microbial taxa (e.g., *Staphylococcus*, *Bacillus*, *Anaerostipes*) may vary across the AD continuum and have been associated with cognitive performance and brain-related changes [13].

Both clinical and preclinical studies report microbial dysbiosis in AD, commonly characterized by reduced diversity and shifts in microbial composition [2, 10, 11, 14–16]. In particular, alterations in specific bacterial taxa have been observed, including increases in pro-inflammatory species such as *Escherichia coli *(*E.coli*)/*Shigella* and *Pseudomonas aeruginosa*, alongside reductions in beneficial microbes such as *Eubacterium rectale/hallii*, and *Faecalibacterium prausnitzii* [17, 18]. These compositional changes are broadly associated with inflammatory responses, metabolic imbalance, and disruptions in gut-brain communication [19, 20].

Beyond compositional changes, microbiota-derived metabolites and microbial components are increasingly recognized as key mediators of gut-brain interactions [21–25]. Among these, short-chain fatty acids (SCFAs), bile acids (BAs), and lipopolysaccharides (LPS) have been implicated in regulating neuroinflammatory responses, blood–brain barrier (BBB) integrity, and neuronal function [26–31]. Collectively, these factors have been associated with inflammatory and metabolic pathways implicated in amyloid pathology and neurodegeneration [32–36]. In particular, LPS derived from gram-negative bacteria has been linked to neuroinflammation and cognitive impairment in AD, although its effects may be context-dependent [35, 37–41].

In parallel with the pro-inflammatory actions of LPS, microbiota-derived metabolites such as SCFAs and BAs contribute to immune and metabolic regulation [42]. SCFA-producing bacteria, including *Faecalibacterium prausnitzii*, *Lachnospiraceae*, and *Eubacterium rectale*, have been associated with anti-inflammatory functions and maintenance of gut barrier integrity, whereas reduced SCFA levels have been linked to microbial dysbiosis and disease severity in AD [43–50]. Similarly, alterations in BA metabolism, influenced by microbial composition such as reduced *Clostridium* and *Bifidobacterium*, have been associated with AD-related biomarkers including amyloid-beta (Aβ) and tau, as well as neuroinflammatory processes [51–56]. Notably, certain BA derivatives such as tauroursodeoxycholic acid (TUDCA), have demonstrated neuroprotective effects in experimental models [57].

Collectively, these findings highlight a complex interplay between gut microbial composition, microbial metabolites, and host neuroimmune signaling in AD [58]. Microbial-derived components, including LPS, can activate immune responses and contribute to pathways associated with synaptic dysfunction and amyloid accumulation [59–61], while reductions in beneficial SCFA-producing bacteria such as *Faecalibacterium prausnitzii* and *Roseburia hominis* may impair immune regulation [62, 63]. Beyond immune modulation, microbiota-derived metabolites also influence neurotrophic signaling, with reduced SCFA levels associated with impaired neuronal function and reduced expression of neurotrophic factors such as brain-derived neurotrophic factor (BDNF) [64–66].

Together, these findings support the concept of a multidirectional “gut-brain-immune axis,” in which microbial composition and function influence neuroinflammatory and neurotrophic signaling networks [67]. This framework provides a basis for exploring microbiota-targeted therapeutic strategies aimed at modulating neuroinflammation and promoting synaptic resilience in neurodegenerative disorders. However, despite growing evidence, the field remains fragmented, with many studies focusing on either descriptive microbial changes or isolated mechanisms rather than integrating both.

Therefore, this systematic review aims to identify specific microbial species associated with AD and to clarify the mechanisms through which they influence disease pathophysiology, including interactions with the immune system, BBB integrity, and Aβ aggregation. By integrating microbial composition with associated metabolites (such as SCFAs and BAs), inflammatory mediators, and microbial components like LPS, this review provides a more cohesive mechanistic framework for understanding the role of the gut-brain axis in AD. This integrative approach aims to bridge the gap between descriptive and mechanistic studies and to support the development of microbiota-targeted therapeutic strategies.

## Methods

### Compliance with PRISMA Guidelines

This systematic review was performed following the Preferred Reporting Items for Systematic Review and Meta-Analysis (PRISMA) 2020 guidelines [68]. The study protocol was structured using the Population, Intervention, Comparators, Outcome, and Study Design (PICOS) framework to ensure methodological transparency [69].

### Eligibility Criteria

Studies were included if they involved human participants clinically diagnosed with AD of any severity, age, or gender, according to established diagnostic frameworks such as the National Institute on Aging–Alzheimer’s Association (NIA–AA) or the Diagnostic and Statistical Manual of Mental Disorders, Fourth or Fifth Edition (DSM-IV/DSM-5). Eligible studies examined the identification, characterization, or modulation of gut microbial taxa or metabolites to explore their association with AD pathology or related mechanisms, including immune responses, BBB integrity, or Aβ aggregation. Comparisons were made with cognitively healthy individuals or non-AD controls without other neurodegenerative diseases. Studies were required to report microbial taxa linked to AD and related metabolic alterations (e.g., SCFAs, BAs, or LPS), as well as immune or inflammatory parameters (e.g., cytokine release, chemokine activity, microglial activation, or neuroinflammation). Eligible study designs included cross-sectional, observational, longitudinal, or experimental cell-culture studies using human-derived samples. Only articles published in English between January 2015, and January 2025 were also considered.

Studies were excluded if they involved only animal models, lacked clearly defined AD diagnostic criteria, included individuals with mixed dementia or other neurodegenerative diseases, or failed to specify microbial taxa or mechanistic links between GM and AD outcomes. Additional exclusions included studies comparing unrelated disorders, those without quantifiable microbiota, metabolites, or immune response data, and non-original works such as case reports, conference abstracts, systematic reviews, commissioned reports, editorials, or organizational papers.

### Search Strategy

A comprehensive search was conducted to identify studies investigating the role of GM in AD. The databases searched included Scopus and PubMed. The search covered publications from January 2015 to January 2025 and was last updated on February 10, 2025. Only peer-reviewed articles published in English were included. The search was structured around two primary objectives.

For objective 1, the search aimed to identify specific microbial species associated with AD. The following search terms and Boolean operators were used:

**Table:** 

(“Alzheimer’s disease” OR “AD”) AND (“gut dysbiosis” OR “gut microbiota” OR “GM” OR “gut microbiome” OR “microbial imbalance” OR “intestinal microbiota” OR “intestinal microbiome” OR “gut flora”) AND (“microbial species identification” OR “bacterial species” OR “pathogenic bacteria”)	

For objective 2, the search focused on mechanistic links between microbial species and neuroinflammation in AD, including immune and metabolic pathways. The following search terms and Boolean operators were used:

**Table:** 

(“Alzheimer’s disease” OR “AD”) AND (“gut dysbiosis” OR “gut microbiota” OR “GM” OR “microbial imbalance” OR “intestinal microbiome” OR “gut flora”) AND (“gut-microbiota metabolites” OR “lipopolysaccharide” OR “LPS” OR “short-chain fatty acids” OR “SCFAs” OR “bile acids” OR “BAs” OR “bacterial amyloids” OR “curli fimbriae” OR “gut-derived β-amyloid”) AND (“neuroinflammation” OR “neuroinflammatory markers” OR “pro-inflammatory cytokines” OR “inflammatory cytokines” OR “inflammasome”) AND (“immune system” OR “immune response” OR “immune cells” OR “microglial cells” OR “astrocytes”) AND (“blood–brain barrier” OR “BBB integrity” OR “BBB dysfunction”) AND (“Aβ aggregation” OR “amyloid-beta” OR “tau pathology” OR “synaptic dysfunction”)	

**Fig. 1:**
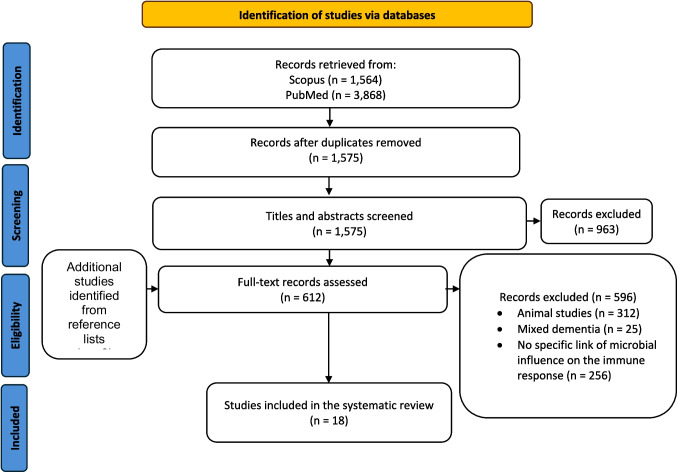
PRISMA flow diagram of selected studies investigating GM alterations and mechanistic links in AD

Search filters were applied to exclude non-human studies, conference abstracts, reviews, and non-original research. Reference lists of all included studies were manually screened for additional eligible articles. The search and screening process are summarized in the PRISMA flow diagram (Fig. 1).

### Study Selection and Data Extraction

The principal investigator screened titles and abstracts for relevance. Duplicate records were removed using EndNote X9. Full-text articles were then assessed against the eligibility criteria. Extracted data included study characteristics (author(s), publication year, sample size, gender and age for AD cases and controls), diagnostic criteria for AD, inclusion/exclusion criteria, sample type, extraction method, sequencing platform, GM composition, metabolite changes, cytokines/chemokines, inflammatory biomarkers, and study strengths and limitations.

### Quality Appraisal

**Fig. 2:**
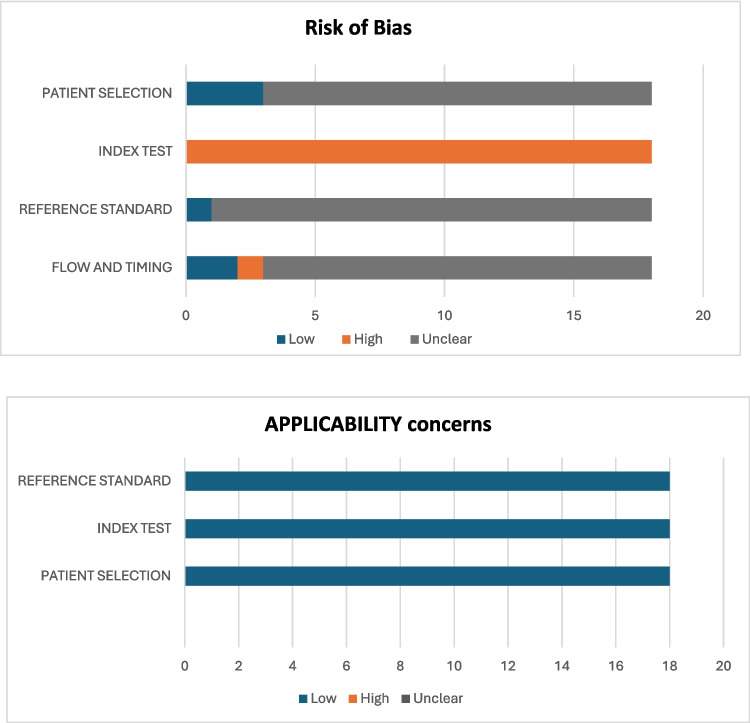
Risk of bias and applicability concerns assessment of the included studies

The Quality Assessment of Diagnostic Accuracy Studies (QUADAS-2) tool was used to evaluate the risk of bias and applicability across four domains: patient selection, index test, reference standard, and flow/timing [70]. The “index test” referred to microbial identification methods, while the “reference standard” was the clinical criteria for AD diagnosis. Each domain was assessed for risk of bias and applicability concerns (Fig. 2).

### Synthesis

Due to methodological and analytical heterogeneity among studies, a narrative synthesis approach was adopted. Thematic analysis was used to categorize findings into two main themes: (1) identification of specific GM compositions in AD patients versus controls (anti-inflammatory bacteria, pro-inflammatory bacteria, and bacteria with both properties*)*, and (2) microbial mechanisms affecting AD pathophysiology (LPS and metabolism, SCFAs and metabolism, BAs and metabolism, cytokine and chemokine profiles, and inflammatory signaling).

## Results

The initial search retrieved 5432 articles. After removing 3857 duplicates, titles and abstracts were screened, leading to the exclusion of studies that failed to meet the inclusion criteria or were irrelevant. The remaining 612 studies were thoroughly assessed, with 16 meeting the inclusion criteria and included in the analysis. Additionally, two studies identified through reference lists were also included. As a result, the systematic review comprised 18 studies. Figure 1 illustrates the selection process and reasons for exclusion.

### Risk of Bias Assessment

Among the included studies, only three [15, 48, 71] employed consecutive sampling methods, while the remaining studies provided insufficient detail on sampling techniques, resulting in an unclear risk of bias in the patient section domain. Regarding the index test, the overall risk of bias was high. Although all studies utilized validated diagnostic tests, none reported implementing blinding during diagnosis. Additionally, three studies [48, 51, 72] did not pre-specify the threshold used for interpretation. The risk of bias concerning the reference standard was unclear, as only one study [73] confirmed blinding to index test results. Similarly, in the flow and timing domain, two studies [74, 75] reported the time interval between the index test and reference standard, leaving the risk of bias unclear. One study [76] posed a high risk of bias in this domain, as not all patients received the reference standard. In terms of applicability, all studies included participants aligned with the research question. The use of reliable diagnostic criteria and appropriate index tests resulted in low applicability concerns (Fig. 2).

### Overview of Features of Included Studies

The studies included in the analysis were published between 2016 and 2023. Study populations ranged from 42 to 302, including 18 to 100 AD cases (mean age 72.9) and 13 to 115 controls (mean age 71.5). In total, the AD group comprised 188 females and 189 males, while the control group included 227 females and 217 males, indicating a slight female predominance across both groups. AD patients were diagnosed and differentiated from controls using established diagnostic criteria. Feces samples were the most commonly used sample type across the studies. DNA extraction was primarily conducted utilizing the QIAamp DNA Stool Mini kit and the PowerSoil DNA Isolation kit. The Illumina MiSeq platform was the most frequently used sequencing platform. A summarized table of study characteristics is presented in Table 1.

**Table 1:** An overview of the included studies

Study	Year	*N*	Age (mean/SD)	Gender	Diagnostic criteria for AD	Sample type	Extraction method	Sequencing platform
Zhan et al. [77]	2016	42 (24 AD, 18 controls)	AD: 76.9/1.9 controls: 80.9/1.1	Male AD: 9 controls: 10	CERAD criteria, Braak staging	PCR analysis, brain tissue	PureLink Genomic DNA kit	BigDye Terminator v3.1 Cycle Sequencing kit, CleanSEQ
Vogt et al. [8]	2017	50 (25 AD, 25 controls)	AD: 71.3/7.3 controls: 69.3/7.5	Female AD: 17 controls: 18	NINDS/ADRDA	feces	NucleoSpin Gel, PCR Clean-up kit	Illumina MiSeq
Zhuang et al. [51]	2018	86 (43 AD, 43 controls)	AD: 70.12/8.78 controls: 69.72/9.24	Female AD: 20 controls: 20	MMSE (Chinese version), ADL, CDR, fuld object memory evaluation, rapid verbal retrieve, PET	feces	PowerSoil kit	Illumina MiSeq
Haran et al. [75]	2019	108 (24 AD, 51 controls)	AD: 84.7/8.1 controls: 83/10.2	Male AD: 4 controls: 8	CDR	stool	PowerMag Soil DNA Isolation kit	Illumina NextSeq 500
Liu et al. [71]	2019	97 (33 AD, 32 controls)	AD: 74.85/11.37 controls: 76.88/9.35	Female AD: 14 controls: 16	DSM-IV, NINCDS-ADRDA, CDR, MMSE, MoCA (Beijing version)	feces	QUIAGEN DNA extraction kit	Illumina MiSeq
Li et al. [72]	2019	90 (30 AD, 30 controls)	AD: 66.3/5.1 controls: 63.9/5.1	Male AD: 15 controls: 13	NIA-AA, MMSE	feces blood	ND	ND
Guo et al. [73]	2021	56 (18 AD, 18 controls)	AD: 63.5/4.7 controls: 64.2/4.7	Male AD: 2 controls: 4	NIA-AA, MMSE MoCA, ADL, CDR, ADAS-Cog	feces	PowerSoil DNA Isolation kit (MOBIO Laboratories)	Illumina Miseq/Microseq
Ling et al. [78]	2021	171 (100 AD, 71 controls)	AD: 74.14/9.21 controls: 73.11/7.75	Male AD:43 controls: 35	MMSE (Chinese version), NINCDS-ADRDA, instrumental Barthel activities of daily living, MRI	feces	QIAamp DNA Stool mini kit	Illumina Miseq
Wu et al. [48]	2021	77 (27 AD, 28 controls)	AD: 74.15/11.16 controls: 74.25/9.03	Male AD: 15 controls: 14	MMSE, MoCA (Beijing version), DSM-IV, NINCDS-ADRDA, CDR, MRI	feces serum	ND	ND
Verhaar et al. [79]	2021	169 (33 AD, 115 controls)	AD: 66/8 controls: 62/8	Female AD: 15 controls: 50	MMSE, CSF (Aβ, p-tau), PET, MRI	feces	PowerSoil DNA Isolation kit	Illumina MiSeq
Zhu et al. [74]	2022	302 (83 AD, 94 controls)	AD: 71.8/8.3 controls: 74.3/10.6	Female AD: 53 controls: 58	NIA-AA	feces	E.Z.N.A. Soil DNA kit	Illumina MiSeq PE300
Laske et al. [13]	2022	175 (75 AD, 100 controls)	AD: 68.6/8.6 controls: 71/4.4	Male AD: 41 controls: 46	NIA-AA, CDR, MMSE	CSF stool blood	QIAamp DNA Blood Maxi kit	Illumina MiSeq
Jeong et al. [80]	2022	98 (38 AD, 50 controls)	AD: 77.13/9.34 controls: 61.9/9.13	Male AD: 12 controls: 16	NINCDS-ADRDA	feces	QIAamp PowerFecal Pro DNA kit	Illumina NovaSeq 6000
Yildirim et al. [76]	2022	125 (47 AD, 51 controls)	AD: 71.4/5.1 controls: 67/5.3	Female AD: 23 controls: 23	NIA-AA, MMSE, CDR, CSF Αβ42/Αβ40 ratio, p-tau, p-tau/Aβ42 ratio	stool CSF	QIAamp DNA Stool Mini kit	Illumina MiSeq
Wang et al. [81]	2022	60 (30 AD, 30 controls)	AD: 79.80/8.55 controls: 78.23/9.71	Male AD: 24 controls: 22	MMSE, MoCA, IWG-2 (2014)	feces blood	Stool DNA kit (Omega, USA)	Illumina MiSeq 250
Kaiyrlykyzy et al. [82]	2022	84 (41 AD, 43 controls)	AD: 68 controls: 68	Female AD: 30 controls: 35	MMSE, CDT, NINCDS-ADRDA	feces blood	QIAamp DNA Stool Mini kit (Qiagen, 51504)	Illumina
Marizzoni et al. [83]	2023	85 (34 AD, 13 controls)	AD: 70.8/6.1 controls: 69.6/7.1	Female AD: 16 controls: 7	MMSE, ADAS-Cog	stool blood	QIAamp DNA Stool Mini kit	Illumina MiSeq
Ferreiro et al. [84]	2023	164 (49 AD, 115 controls)	AD: 78.96/4.51 controls: 77.02/5.80	Male AD: 24 controls: 49	CDR, PET, CSF Aβ42/Αβ40 ratio	stool	DNeasy PowerSoil Pro kit	Illumina NovaSeq 6000

*AD* Alzheimer’s disease, *CERAD* Consortium to Establish a Registry for AD, *PCR* polymerase chain reaction, *NINDS/ADRDA* National Institute of Neurological Disorders and Stroke/Alzheimer’s Disease and Related Disorders Association, *MMSE* Mini-Mental State Examination, *ADL* activities of daily living, *CDR* clinical dementia rating, *PET* Positron emission tomography, *DSM-IV* Diagnostic and Statistical Manual of Mental Disorders, fourth edition; *NINCDS-ADRDA* National Institute of Neurological and Communicative Disorders and Stroke-Alzheimer’s Disease and Related Disorders Association, *MoCA* Montreal Cognitive Assessment, *NIA-AA* National Institute on Aging and Alzheimer’s Association, *ADAS-Cog* Alzheimer’s Disease Assessment Scale-Cognitive subscale, *MRI* magnetic resonance imaging, *CSF* cerebrospinal fluid, *CDT* clock drawing test; *IWG-2 (2014)* International Working Group on the Diagnosis of Alzheimer’s Disease-2nd Meeting (2014), *ND* not determined

## Identification of Specific GM Compositions in Patients with AD Vs Controls

### Anti-Inflammatory Bacteria

Within the phylum *Bacteroidota*, several taxa commonly described as anti-inflammatory showed variable associations with AD. *Bacteroides fragilis*, *Bacteroides dorei*, *Odoribacter splanchnicus* and *Barnesiella species* were reported as more abundant in one study [75], whereas other studies observed decreased abundance of *Barnesiella species* [72] and *Odoribacter splanchnicus* [79].

Within the phylum *Bacillota* (*Firmicutes*), a predominantly reduced abundance of taxa previously described as having anti-inflammatory functions was reported, including *Eubacteriales* (*Clostridiales*) [48], *Marvinbryantia* species [79], *Blautia* [48], *Blautia hanseii*, *Ruminococcus bicirculans* [75]*, Faecalibacterium* [76, 78], *Faecalibacterium prausnitzii* [75, 84], and *Butyricicoccus* [78, 79].

Conversely, increased abundance was reported for taxa such as *Oscillibacter* [74], *Anaerostipes hadrus* [84], *Christensenellaceae* [82], *Blautia hydrogenotrophica* [80], *Dorea* [72], *Dorea formicigenerans* [84], *Lactobacillales* [51], *Carnobacteriaceae* [74], *Phascolarctobacterium* [8], and *Dialister* OTU140 [82], indicating complex microbial interactions. Notably, *Blautia hydrogenotrophica* abundance was negatively correlated with cognitive performance across AD subgroups [80]. Additional bacterial taxa are presented in Table 2.

**Table 2:**
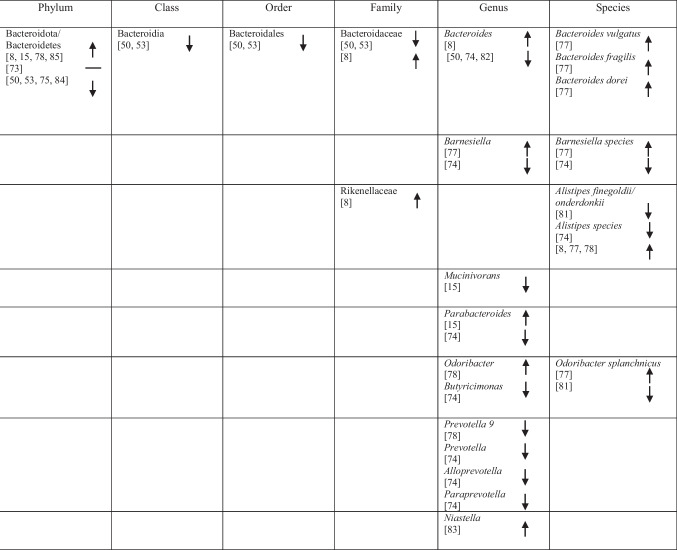

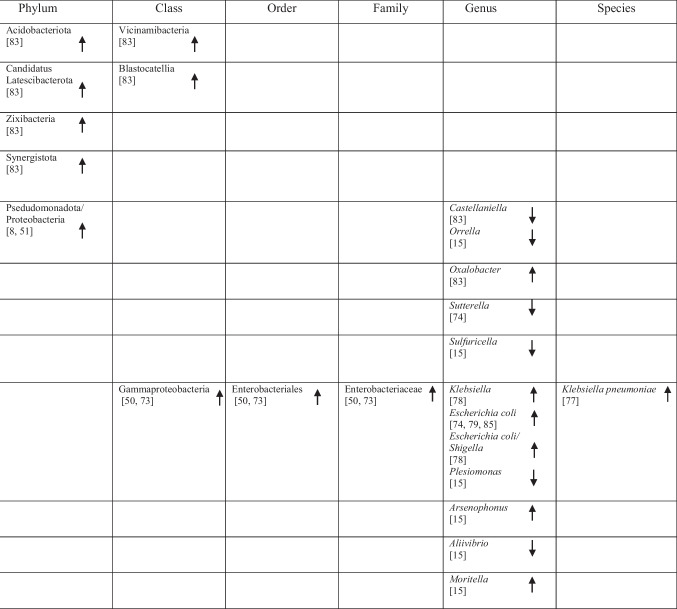

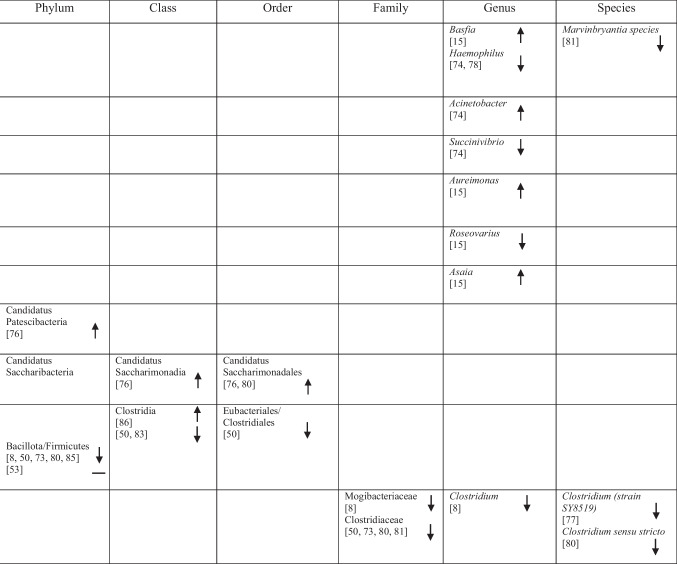

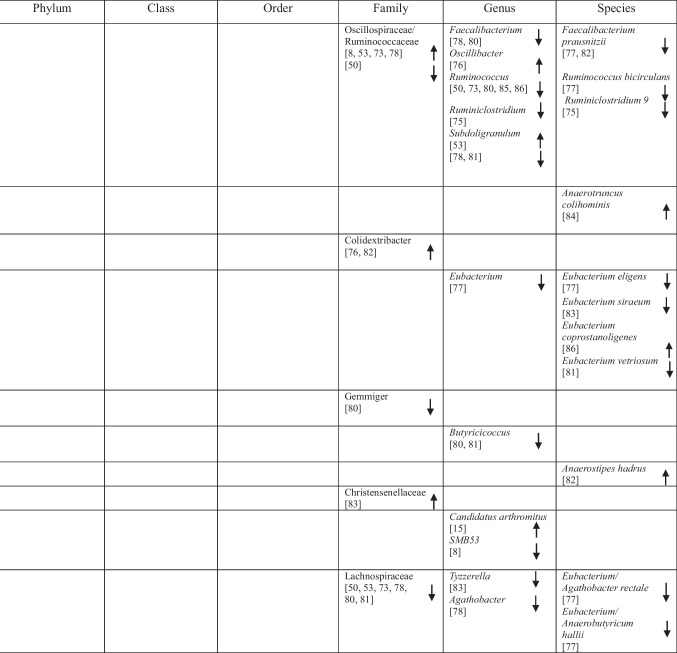

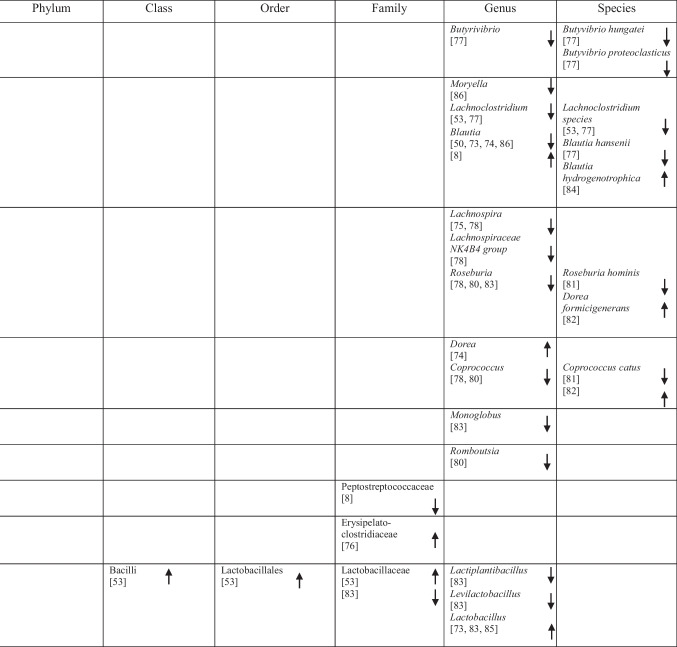

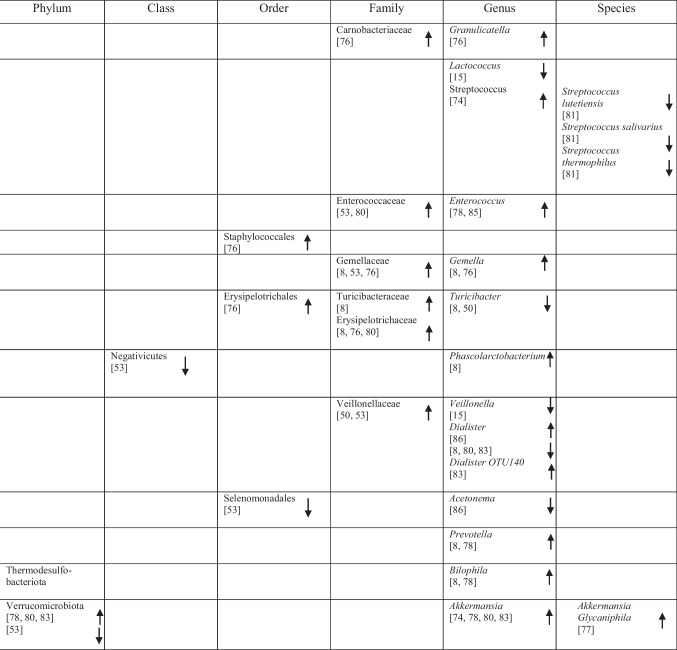

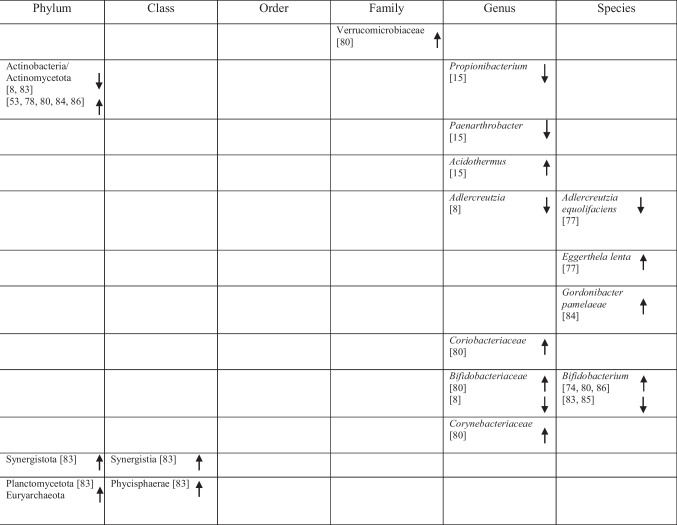
Abundance of GM in AD as reported in the included studies

### Pro-Inflammatory Bacteria

Taxa commonly associated with pro-inflammatory profiles also showed heterogeneous patterns across studies. Within *Bacteroidota, Bacteroides vulgatus* was reported to be elevated in AD [75], whereas within *Euryarchaeota*, *Methanosphaera* stadtmanae was decreased [84]. Likewise, five studies on *Pseudomonadota* (formerly *Proteobacteria*) reported increased levels of *Klebsiella* [76], *Klebsiella pneumoniae* [75], *E.* coli [72, 77, 81], while decreased levels of *Sulfuricella* [15], *Plesiomonas* [15], *Succinivibrio* [72], and *Roseovarius* [15] were observed. Enrichment of *Proteobacteria* in AD feces co-occurred with adverse AD biomarker profiles, linking these gram-negative bacteria to greater disease severity [8, 49].

Within *Bacillota,* reduced abundance was observed for *Clostridium *sensu stricto [78], *Tyzzerella*, and *Monoglobus* [82], whereas *Anaerotruncus colihominis* [80] and *Colidextribacter* were reported as increased [74, 84]. Additional taxa are listed in Table 2.

### Bacteria with Both Properties

Several taxa demonstrated variable associations across studies, reflecting potential context-dependent roles [85]. Increases in *Rikenellaceae* [8], *Aureimonas* [15], *Streptococcus* [72], *Enterococcus* [76, 81], and *Staphylococcales* [74] were reported, while *Mucinivorans* and *Aliivibrio* [15], *Prevotella* [72], *Negativicutes*, and *Selenomonadales* [51] were reduced. *Clostridia* demonstrated inconsistent patterns, with increased abundance reported in one study [83] and decreased abundance in others [48, 82]. A detailed overview of these taxa is provided in Table 2.

## Microbial Mechanisms Affecting AD Pathophysiology

### LPS and Metabolism

Two studies reported associations between LPS levels and gut microbial composition in AD. Reduced abundance of *Moryella* was reported in association with elevated LPS levels [83]. In another study, increased concentrations of *E. coli* were associated with higher LPS concentrations in both grey and white matter of AD brains. Elevated LPS levels were also reported in association with AD-related biomarkers, including amyloid plaque burden and tau pathology, and measures of cognitive decline [77].

### Scfas and Metabolism

Most studies reported reduced SCFAs, including acetate, propionate, and butyrate, in AD patients.

These reductions were observed alongside decreased abundance of multiple taxa involved in SCFA production, including members of *Bacillota*, *Clostridia*, *Eubacteriales*, *Clostridiaceae*, *Lachnospiraceae*, *Oscillospiraceae* (formerly *Ruminococcaceae*), *Ruminococcus*, *Bacteroides*, *Bacteroidaceae*, *Bacteroidales*, *Blautia*, *Bacteroidia* [48], *Subdoligranulum* [76, 79], *Eubacterium vetriosum*, *Roseburia hominis*, *Marvinbryantia* species, *Streptococcus lutetiensis*, *Streptococcus salivarius*, *Streptococcus thermophilus*, *Alistipes finegoldii/onderdonkii, Odoribacter splanchnicus*, *Coprococcus catus* [79], *Blautia hanseii*, *Eubacterium eligens*, *Eubacterium hallii*, *Eubacterium rectale*, *Butyvibrio hungatei*, *Butyvibrio proteoclasticus*, *Clostridium* species strain SY8519, *Faecalibacterium prausnitzii* and *Ruminococcus bicirculans* [75], *Faecalibacterium*, *Roseburia*, *Gemmiger*, *Coprococcus*, and *Butyricicoccus* [78].

Several of these taxa, particularly *Faecalibacterium*, *Roseburia*, *Eubacterium*, and *Ruminococcus*, were reported to correlate with cognitive test performance and clinical severity [48, 80]. Reduced SCFA levels were also reported in association with markers of gut barrier dysfunction and systemic inflammation, which were discussed in the original studies as potential contributors to neuroinflammatory processes and amyloid pathology [48, 49]. These metabolic alterations co-occurred with increased inflammatory metabolites and altered host-microbe interaction signals in individuals with lower cognitive performance [48]. In addition, altered SCFA-related pathways, including depletion of butanoate and propanoate biosynthesis genes, were reported in individuals with greater cognitive impairment [49]. However, increased levels of *Enterobacteriaceae*, *Enterobacteriales*, *Veillonellaceae*, and *Gammaproteobacteria* [48] were also associated with disrupted metabolic function.

Some of these bacteria were reported to correlate with AD-related biomarkers, including Aβ [79], and with expression levels of P-glycoprotein (P-gp) [75]. In addition, taxon-metabolite associations were described, with *Bacillota* correlating with formic acid and DL-5-methoxytryptophol (DL-5-MTP), and *Clostridia* and *Eubacteriales* correlating with formic acid, DL-5-MTP, and 2-methylbutyric acid [48]. *Ruminococcus* was associated with Indole-2-carboxylic acid, and 3-(2-Hydroxyethyl)indole, while *Lachnospiraceae* correlated with 2-Methylbutyric acid [48]. This study also measured tryptophan-derived indole metabolites and reported taxon-metabolite correlations, including associations between *Ruminococcus* and indole-type derivatives, which differed between controls and the AD group [48]. Conversely, *Enterobacteriaceae*, *Enterobacteriales*, and *Gammaproteobacteria* showed negative correlations with formic acid, while *Veillonellaceae* was negatively correlated with Indole-2-carboxylic acid [48].

### Bas and Metabolism

One study reported that increased *Veillonellaceae* and decreased *Ruminococcus* abundance were associated with reduced levels of lithocholic acid (LCA), a secondary bile acid [48]. Altered BA profiles were further correlated with poorer memory and executive performance among AD participants. These findings were reported in association with microbial composition, BA metabolism, and cognitive performance via neuroinflammatory and mitochondrial pathways, although causal relationships cannot be established from the available data [48].

### Cytokine and Chemokine Profiles

Alterations in GM composition were reported in association with changes in cytokine and chemokine levels. Reduced abundance of taxa such as *Acetonema*, *Blautia*, and *Ruminococcus* was associated with elevated levels of pro-inflammatory cytokines, including Interleukin-6 (IL-6), Interleukin-1 beta (IL-1β), and tumor necrosis factor-alpha (TNF-α), with IL-6 and TNF-α contributing to endothelial damage [83]. Similarly, decreased levels of *Gemmiger*, *Roseburia*, *Faecalibacterium* and *Coprococcus* correlated with increased TNF-α and the chemokine IP-10 [78]. Associations involving *Bifidobacterium* were inconsistent, with some studies reporting positive correlations with pro-inflammatory cytokines (IL-1β, IL-6, TNF-α, Interleukin-8 (IL-8)) alongside reduced Interleukin-10 (IL-10) [83], while others reported that higher abundance of *Bifidobacterium* and *Bifidobacteriaceae* was associated with lower IL-8 levels [78]. These cytokine profiles were reported alongside poorer MMSE and MoCA scores, indicating inflammatory signaling as a mediator between gut dysbiosis and cognitive impairment [78, 83]. Additional associations were observed between *Clostridia* and IL-1β and IL-6 [83], and between *Akkermansia*, which was positively correlated with IP-10 and negatively with Interferon-gamma (INF-γ) [78].

In contrast, higher levels of certain taxa typically described as anti-inflammatory, such as the *Eubacterium coprostanoligenes* group, were also associated with elevated pro-inflammatory cytokines [83]. Likewise, elevated abundance of species within the *Corynebacteriaceae* and *Enterococcaceae* families [78], as well as *Enterococcus* and *E. coli* [81], were linked to elevated TNF-α. However, no significant associations were observed between increased *Enterococcus* and *E. coli* abundance and IL-1β, IL-6, or IL-8 levels [81].

### Inflammatory Signaling

Two studies reported associations between chitinase-3-like protein 1 (YKL-40) levels and GM composition. Reduced abundance of the genus *Turicibacter* was associated with higher YKL-40 levels [8, 48]. Similarly, decreased abundance of *SMB53* was correlated with elevated YKL-40 levels [8].

In contrast, the influence of *Bacteroides* on YKL-40 levels varied between studies. One study reported reduced *Bacteroides* abundance in association with lower YKL-40 levels [48], whereas another reported increased *Bacteroides* abundance in AD patients alongside higher YKL-40 levels, which were also correlated with increased p-tau and p-tau/Aβ42 concentrations in CSF [8]. Elevated YKL-40 levels were further reported in association with poorer cognitive performance in the same study [8]. An overview of microbiota and their reported associations with AD-related pathways is presented in Table 3.

**Table 3:**
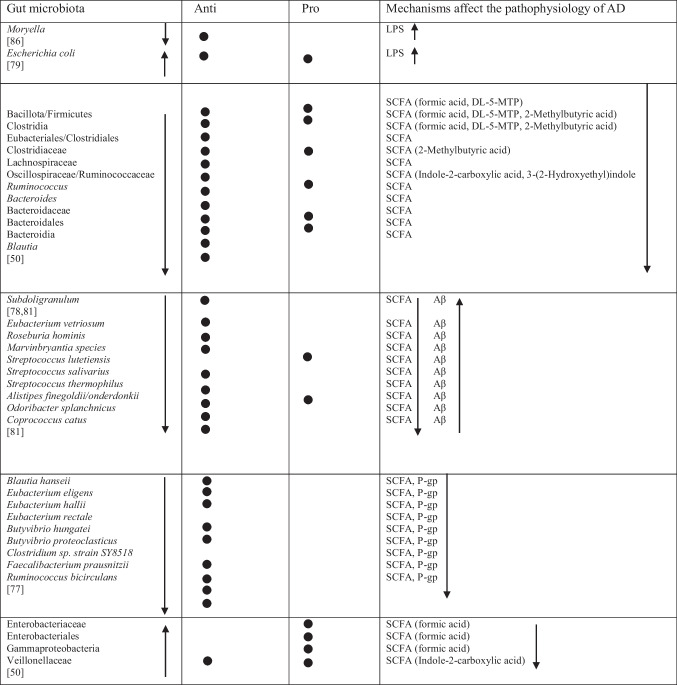

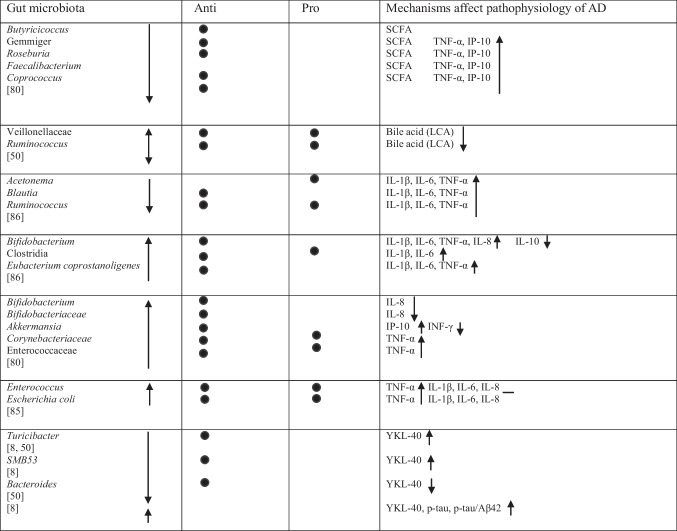
An overview of GM and their mechanisms in AD as reported in the included studies

*Anti* anti-inflammatory, *Pro* pro-inflammatory, *GM* gut microbiota, *AD* Alzheimer’s disease, *Aβ* amyloid-beta, *LPS* lipopolysaccharide, *SCFA* short-chain fatty acids, *BA* bile acid, *p-gp* p-glycoprotein, *YKL-40* chitinase-3-like protein 1, *DL-5-MTP* DL-5-methoxytryptophol, *LCA* lithocholic acid, *IL-1β* interleukin-1 beta, *IL-6* interleukin-6, *IL-8* interleukin-8, *IL-10* interleukin-10, *TNF-α* tumor necrosis factor-alpha, *IP-10* interferon gamma-induced protein 10, *inf-γ* interferon-gamma

## Discussion

### GM Composition in AD Vs Controls

#### Anti-Inflammatory Bacteria

Across studies, no consistent taxonomic signature of “protective” microbiota in AD is observed. Variability in taxa such as *Actinobacteria* and *Bifidobacterium* across cohorts [8, 51, 72, 78, 81, 82] suggests that host-related factors, including diet, geography, and disease stage, strongly influence microbiota composition [86]. This heterogeneity limits the interpretability of individual taxa and indicates that microbial function, rather than taxonomic composition, is the more relevant determinant of AD-associated alterations.

Despite these inconsistencies, converging evidence supports a functional role for specific taxa. For example, *Bifidobacterium lactis* Probio-M8 improves cognition and reduces Aβ pathology in APP/PS1 models [87], while reduced *Adlercreutzia equolifaciens* may increase susceptibility to oxidative stress and endotoxin-driven inflammation [8, 88]. Similarly, reductions in SCFA-producing taxa within the *Bacillota* phylum, including *Lactobacillaceae*, *Ruminiclostridium*, and *Coprococcus*, are frequently associated with impaired metabolic and neuroimmune regulation [73, 89–93], although inconsistencies remain [51, 76, 78, 82, 92, 94], suggesting that microbial function, rather than taxonomic presence alone, may be more relevant to disease progression.

Notably, key SCFA-producing genera such as *Coprococcus*, *Roseburia*, and *Faecalibacterium*, often considered neuroprotective, show variable associations across AD cohorts and disease stages [76, 78, 82, 94, 95]. In parallel, *Oscillospiraceae* has been linked to metabolic dysfunction and insulin resistance [96], a known risk factor for AD [96–104]. Similarly, *Akkermansia* despite its role in mucosal homeostasis, has been associated with impaired gut barrier integrity and poorer cognitive outcomes in AD [72, 75, 76, 78, 82, 92, 105, 106].

Within the *Bacteroidota* phylum, genera such as *Barnesiella* and *Odoribacter* also show inconsistent associations with AD [107, 108]. *Barnesiella* has been linked to both cognitive decline and inflammatory dysregulation [107], while lower levels correspond to increased cerebral small vessel disease burden [108]. Although Mendelian randomization suggests a protective role in cognition [109], these findings remain inconsistent across cohorts [72, 75], limiting firm conclusions regarding causality. Similarly, *Odoribacter splanchnicus* has been associated with AD in experimental and clinical studies [50, 75, 76, 79, 110], but several associations are lost after adjustment for confounders [79], further highlighting methodological sensitivity.

Collectively, these findings indicate that taxonomic associations are highly context-dependent and sensitive to cohort and analytical variation, reinforcing the need for functionally informed interpretations of microbiome alterations in AD.

### Pro-Inflammatory Bacteria

In contrast, enrichment of pro-inflammatory taxa represents a more consistent feature of AD-associated dysbiosis. Increased abundance of gram-negative bacteria, including *Klebsiella pneumoniae,* is observed in prodromal AD and is associated with neuroinflammation and behavioral deficits in transgenic models [94, 111]. Similarly, *Ε. coli* contributes to disease progression through production of extracellular amyloids (e.g., CsgA) and activation of immune pathways, with evidence of plaque colocalization and neuroinflammatory effects in AD brains [72, 73, 76, 77, 81, 112].

Other taxa, including *Eggerthella lenta* (*E. lenta)* and *Candidatus Arthromitus* (*C. Arthromitus*), demonstrate context-dependent pathogenicity. While *E. lenta* may generate beneficial metabolites under physiological conditions, its enrichment in AD and MCI [75, 113] suggests a shift toward detrimental metabolic activity. Likewise, increased *C. Arthromitus* has been associated with immune dysregulation and inflammatory responses relevant to AD [15, 114–118].

Interestingly, members of *Clostridium *sensu stricto exhibit dual roles, with some species contributing to immune regulation and others associated with pathogenicity. Reduced abundance has been linked to worse cognitive outcomes [78, 119], whereas preservation may confer neuroprotective effects [120–122]. Overall, these findings support a model in which pro-inflammatory taxa may contribute to AD progression through sustained immune activation and disruption of gut-brain homeostasis.

### Bacteria with Both Properties

Several taxa exhibit both protective and pathogenic properties depending on host and disease context. *Ruminococcus*, for example, is reduced in aMCI and AD and is implicated in metabolic regulation and gut barrier integrity [48, 71, 78, 81, 83, 123–126]. Given its role in propionate production and metabolic homeostasis [123], its depletion may contribute to increased intestinal permeability and translocation of microbial products, including amyloids [124]. Experimental evidence further supports its functional relevance, as probiotic intervention has been shown to restore *Ruminococcus* abundance in models of gut injury [127], suggesting a role in maintaining microbial ecosystem stability rather than representing a fixed disease marker.

Similarly, members of the genus *Eubacterium*, including *Eubacterium nodatum* (*E. nodatum*) and *Eubacterium fissicatena*, are generally reduced in AD and negatively associated with AD disease status [128]. As butyrate-producing taxa [129], their depletion aligns with reduced SCFA-related metabolic capacity and decreased expression of butyrate-associated genes in AD [75]. Nevertheless, some species, particularly *E. nodatum*, have also been linked to neurodegenerative processes, indicating strain-specific effects [128, 129].

Likewise, *Peptostreptococcaceae* supports intestinal homeostasis [130], and its reduction has been associated with poorer cognitive outcomes, with lower levels observed in AD patients [8]. In contrast, *Prevotella* is frequently elevated in aging-related neurological disorders. For example, an Egyptian cohort revealed increased *Prevotella* abundance negatively correlated with cognitive performance and positively with disease duration [131]. Additional studies report elevated *Prevotella* levels in AD [73, 82].

These findings indicate that microbial effects are not uniformly beneficial or harmful but are shaped by functional context, host metabolism, and disease stage, highlighting the limitations of taxonomic classification alone.

### Mechanisms by Which Microbes Affect AD Pathophysiology

#### LPS and Metabolism

**Fig. 3:**
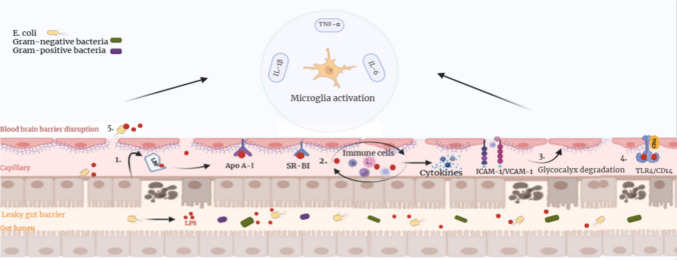
LPS-mediated mechanisms contributing to BBB disruption and neuroinflammation in AD. A leaky barrier permits LPS translocation into the bloodstream. (1) LPS binds to LBP and crosses the BBB via ApoA-I and SR-BI receptors. (2) It induces cytokine release and upregulates ICAM-1/VCAM-1, facilitating immune cell infiltration. (3) TNF-α production leads to glycocalyx degradation and increased BBB permeability. (4) LPS directly activates endothelial TLR4/CD14 signaling. (5) Gram-negative bacteria cross the BBB, delivering LPS into the brain. These processes trigger microglial activation and elevated IL-1β, IL-6, and TNF-α expression, promoting AD-related neuroinflammation

The findings of this review support a potential role for lipopolysaccharides (LPS) in linking gut dysbiosis to neuroinflammation in AD. Increased abundance of gram-negative bacteria, including *E. coli*, is associated with elevated LPS levels and systemic inflammation [32, 132], with LPS and bacterial components (e.g., K99) detected in AD brain tissue [77]. LPS has been proposed to contribute to AD-related processes through several interconnected mechanisms (Fig. 3):

1. *Crossing the BBB:* LPS binds to lipopolysaccharide-binding proteins (LBP) and crosses the BBB via receptors such as apolipoprotein A-I and scavenger receptor class B type I on endothelial cells [133].
2. *Immune cell phagocytosis*: LPS promotes cytokine release and endothelial adhesion molecule expression, facilitating immune cell migration into the brain [134, 135].
3. *Degrading the glycocalyx*: LPS-induced inflammatory mediators, including TNF-α, degrade the endothelial glycocalyx, increasing BBB permeability [136].
4. *Interaction with TLR4/CD14 complex*: LPS activates the TLR4/CD14 complex on endothelial and immune cells, initiating pro-inflammatory signaling [133].
5. *Bacterial translocation*: Gram-negative bacteria may translocate across the BBB, further delivering LPS and exacerbating inflammation [137].

In addition, LPS has been shown to colocalize with Aβ plaques and promotes Aβ accumulation in experimental models [16, 77]. It also impairs Aβ clearance by downregulating LRP-1 and P-glycoprotein (P-gp), disrupting BBB transport mechanisms [138].

At the cellular level, LPS activates the TLR4/MyD88/NF-κB signaling axis, leading to microglial activation, oxidative stress, and neuronal damage [139, 140]. This is accompanied by increased production of pro-inflammatory cytokines, including IL-1β, IL-6, and TNF-α, which contribute to tau pathology and synaptic dysfunction [141]. Activation of the NLRP3 inflammasome further amplifies these responses, promoting chronic neuroinflammation and neurodegeneration [142, 143]. Conversely, reductions in certain gram-positive taxa, including *Moryella*, may exacerbate these effects by weakening gut barrier integrity and limiting anti-inflammatory signaling [81, 83]. Together, these findings highlight LPS as a potential mediator linking gut dysbiosis to neuroinflammatory cascades in AD.

#### Scfas and Metabolism

This review also identifies reduced SCFAs as a consistent feature of AD-associated dysbiosis, likely driven by declines in key SCFA-producing taxa such as *Eubacterium*, *Roseburia*, *Coprococcus*, and *Odoribacter* [79]. Given their roles in maintaining epithelial and BBB integrity, reduced SCFA levels may contribute to barrier dysfunction and systemic inflammation [93, 144–147].

Beyond barrier function, SCFAs also exert neuroprotective effects through epigenetic and neurotrophic mechanisms. Butyrate acts as a histone deacetylase (HDAC) inhibitor, promoting histone acetylation and brain-derived neurotrophic factor (BDNF) expression, thereby supporting synaptic plasticity and memory [66, 148]. Reduced SCFAs may therefore impair BDNF signaling and contribute to synaptic dysfunction and cognitive decline in AD [149]. In addition, SCFAs modulate gut-brain communication via vagal pathways, and their depletion may disrupt neuroimmune and neuroendocrine signaling [41, 150].

Consistent with these functional roles, reduced abundance of SCFA-producing genera such as *Butyricicoccus*, *Gemmiger*, and *Coprococcus* has been associated with increased pro-inflammatory mediators, including TNF-α and IP-10 [78]. Similarly, specific Bacillota species (e.g., *Marvinbryantia* spp. and *R. hominis*) have been linked to increased Aβ deposition [79], further supporting a relationship between impaired SCFA metabolism and AD pathology. Experimental evidence reinforces this association, as butyrate supplementation or administration of *Clostridium butyricum* reduces microglial activation, lowers TNF-α levels, and decreases Aβ accumulation in APP/PS1 models [149]. Mechanistically, butyrate also inhibits histone deacetylation and suppresses NF-κB signaling, thereby reducing neuroinflammatory and amyloidogenic processes [45, 149].

In addition to inflammatory regulation, SCFAs influence amyloid clearance mechanisms through modulation of P-gp expression at the BBB. SCFA-producing taxa such as *Eubacterium rectale* (*E. rectale*) and *Faecalibacterium prausnitzii* (*F. prausnitzii*) have been associated with the maintenance of P-gp expression, whereas their depletion is linked to impaired transport function and increased intestinal and endothelial inflammation [151]. SCFAs regulate P-gp activity via both HDAC inhibition and G-protein coupled receptor (GPR) signaling pathways [152, 153]. In this review, *Faecalibacterium prausnitzii*, *Blautia hanseii* (*B. hanseii*), and* Eubacterium eligens* emerged as taxa associated with P-gp expression, supporting previous findings [75]. Reduced P-gp expression is further associated with gastrointestinal inflammation and increased MRP2 expression, indicating chronic intestinal inflammation [154]. Notably, certain *Bacteroides* species may also modulate P-gp expression, suggesting interactive effects between LPS-mediated inflammation and SCFA depletion [21].

**Fig. 4:**
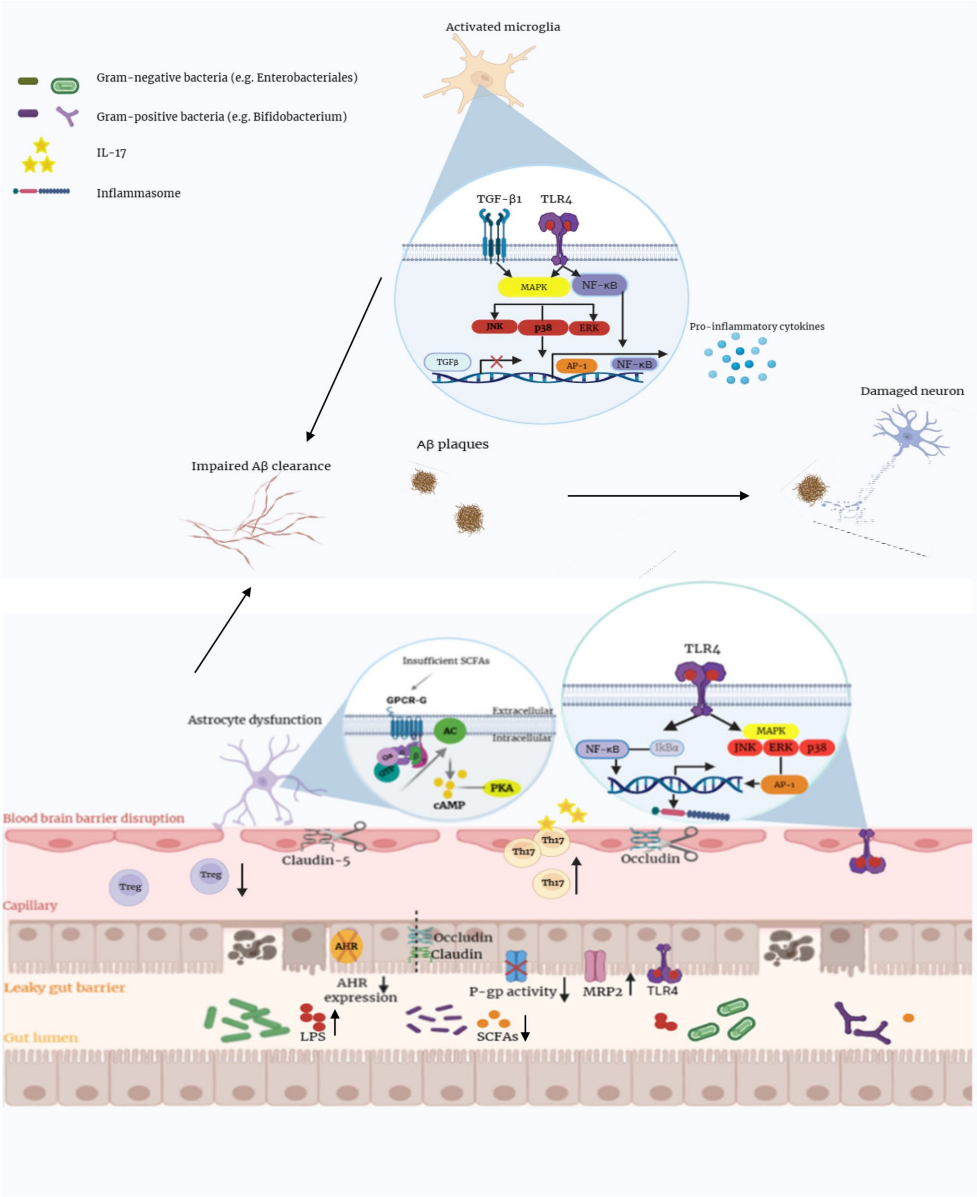
Overview of gut microbiota-mediated mechanisms contributing to AD pathology. Gut dysbiosis leads to a reduction in SCFAs, impairing GPCR-cAMP signaling in astrocytes and weakening their supportive function. This dysfunction, along with the downregulation of tight junction proteins (claudin-5, occludin) and AHR expression compromises both gut and BBB integrity, increasing permeability. LPS from gram-negative bacteria activate TLR4 and downstream inflammatory cascades (NF-κB, MAPKs), promoting cytokine production (e.g., IL-1β, TNF-α) and NLRP3 inflammasome activity. This inflammatory environment shifts immune balance by reducing regulatory T cells (Tregs) and increasing pro-inflammatory Th1/Th17 cells, while also altering gut transporter expression (reduced p—gp, increased MRP2). In the brain, the pro-inflammatory state activates microglia, which in the absence of SCFA-induced IL-4 and M2 polarization, fail to clear Aβ effectively. This contributes to Aβ plaque accumulation, chronic neuroinflammation, neuronal damage, and cognitive decline

In contrast, expansion of gram-negative, LPS-producing bacteria exacerbates systemic inflammation and indirectly contributes to SCFA reduction. Increased abundance of *Enterobacteriales*, *Enterobacteriaceae*, and *Gammaproteobacteria*, is associated with depletion of SCFA-producing taxa such as *B. hanseii*, *E. rectale*, *F. prausnitzii* [75], and *Roseburia hominis* [79], further weakening gut barrier integrity. Supporting this, *Christensenellaceae* has been linked to elevated LPS levels in individuals with subjective cognitive decline (SCD), potentially reflecting early barrier dysfunction [155]. Together, these alterations promote a self-reinforcing cycle in which reduced SCFA production and increased LPS exposure exacerbate intestinal permeability and systemic inflammation. Based on these findings, the mechanisms by which SCFA-producing microbiota influence AD pathology can be summarized as follows (Fig. 4):

1. *Astrocyte dysfunction*: The Gs-cAMP pathway is weakened due to insufficient SCFAs required to activate the odorant receptor Olfr920 in astrocytes [156].
2. *Impaired barrier integrity*: Tight junction proteins (claudin-5, occluding) and receptors such as the hydrocarbon receptor (AHR) are downregulated, weakening the BBB and increasing permeability [26, 157].
3. *Gut inflammation and P-gp/MRP2 shift*: Intestinal inflammation decreases P-gp expression and increases MRP2 expression, disrupting SCFA-mediated anti-inflammatory effects [158].
4. *Pro-inflammatory signaling*: LPS activates pathways such as NK-κB, ERK, and JNK, increasing IL-1β and COX-2 expression [149, 159], while enhanced NLRP3 inflammasome activity and BBB dysfunction [160].
5. *Reduced anti-inflammatory responses*: Activation of pathways such as p38MAPK upregulate pro-inflammatory cytokines (IL-6, TNF-α) while reducing TGF-β1 signaling [161], leading to decreased IL-10 + regulatory T cells and increased Th1/Th17 activity [162–164].
6. *Amyloid plaque formation*: SCFA reduction inhibits IL-4 production and M2 microglial activation, reducing Aβ clearance [161] and promoting amyloidogenic APP processing via β- and γ-secretase activity [165].

#### Bas and Metabolism

Altered BA metabolism represents a key pathway linking gut dysbiosis to AD. Microbial conversion of primary to secondary BAs by taxa such as *Lactobacillus*, *Clostridium* cluster XIVα, *Bifidobacterium*, and *Bacteroides* generates metabolites (e.g., DCA, LCA, UDCA) that regulate host metabolism and inflammation via Farnesoid X receptor (FXR) and Takeda G-protein-coupled receptor 5 (TGR5) signaling [53, 54, 137, 166, 167].

Disruption of these pathways is associated with inflammatory signaling, metabolic dysfunction, and neurodegeneration, partly through NF-κB-mediated suppression of FXR activity [168]. Among secondary BAs, LCA has been strongly linked to neuronal toxicity, mitochondrial dysfunction, and cognitive decline [169–171], with elevated levels observed in both AD patients and experimental models. Similarly, decreased LCA levels were linked to higher levels of anaerobic gram-negative bacteria, such as *Veillonellaceae,* and lower levels of gram-positive species such as *Ruminococcus* [48]. These findings align with reports of elevated LCA in the serum of AD patients [169] and in the brains of AD transgenic mouse models, reinforcing its neurotoxic potential [171]. Associations between conjugated LCA derivatives (e.g., glycolithocholic acid (GLCA), taurolithocholic acid (TLCA)) and CSF p-tau further support a link between BA metabolism and AD pathology [55], underscoring the interplay between microbiota composition, BA conversion and neuroinflammation.

In parallel, dysregulation of tryptophan metabolism shifts metabolic pathways toward production of neurotoxic metabolites, while reducing neuroprotective indole derivatives produced by taxa such as *Clostridium sporogenes* and *Peptostreptococcus* species [172, 173]. Together, these findings highlight BA and tryptophan metabolism as interconnected pathways through which microbial activity influences neuroinflammation and neuronal function in AD.

### Cytokines and Inflammatory Signaling

GM alterations are consistently associated with dysregulated inflammatory signaling in AD, although findings remain heterogeneous. Increased abundance of gram-negative bacteria, particularly *Bacteroides*, correlates with elevated YKL-40 and AD-related biomarkers, including p-tau and Aβ [8, 86]. This association is likely mediated by LPS-driven activation of TLR4/NF-κB pathways, linking microbial changes to amyloid and tau pathology [174, 175].

YKL-40 serves as a key intermediary between peripheral microbial alterations and central neuroinflammation. Its expression reflects astrocyte activation and is further induced by pro-inflammatory cytokines such as TNF-α and IL-1β, with increased levels associated with *Bacteroides* abundance [8, 86, 174, 176]. However, associations with specific taxa (e.g., *Turicibacter*, SMB53) indicate that microbial contributions to inflammation are not uniform [176].

Chronic neuroinflammation in AD is characterized by sustained activation of innate immune pathways, including TLR2/TLR4 signaling and the NLRP3 inflammasome [177]. Elevated cytokines and chemokines, such as IL-8 have been associated with neuronal injury and cognitive decline [178, 179], while downstream effects include tau hyperphosphorylation and synaptic loss mediated by GSK-3β and complement pathways [180, 181].

Interestingly, IL-8 shows a negative correlation with beneficial *Bifidobacterium* species [182], although taxa-specific associations remain inconsistent. While increased *Bifidobacterium* abundance has been linked to reduced IL-8 levels, *Akkermansia* demonstrates mixed relationships, including negative correlations with IFN-γ and cognitive performance [78]. This duality highlights that microbial effects on inflammation are context-dependent and not solely determined by taxonomy. For example, *Akkermansia muciniphila* is associated with beneficial roles under homeostatic conditions, including immune modulation and metabolic regulation [183, 184], yet increased *Akkermansia* abundance in AD patients has been linked to elevated IL-8 levels [83]. Similarly, although *Bifidobacterium* is generally recognized for its probiotic effects, some strains have been associated with pro-inflammatory responses in certain disease conditions, further supporting functional variability within taxa. [83, 185].

Microbiome disruptions and increased intestinal permeability further amplify inflammatory signaling. Elevated IL-1β and NLRP3 levels have been associated with increased *Escherichia/Shigella* abundance and reduced anti-inflammatory *E. rectale* [21], while reduced IL-10 and increased IL-6 contribute to a pro-inflammatory environment [186]. Associations between *E. coli* and *Enterococcus* with TNF-α levels have also been reported, although other cytokine relationships are less consistent, likely reflecting cohort variability [81]. While *Enterococcus* is typically commensal, virulent strains can promote systemic inflammation and blood–brain barrier disruption [81].

Overall, these findings indicate that microbiota-driven inflammatory responses in AD are shaped by taxa-specific and functional interactions rather than general dysbiosis, highlighting the need for mechanistic and targeted approaches to microbiome modulation.

### Limitations

Despite intriguing findings, this review has several limitations. First, substantial heterogeneity across studies, including differences in design, population characteristics, sample size, and diagnostic criteria, limits comparability. Given that definitive AD diagnosis requires post-mortem confirmation, potential misclassification of cases cannot be excluded [187]. In addition, variability in microbiome methodologies, including DNA extraction protocols and sequencing platforms, likely contributed to inconsistent results across studies.

Second, although associations between GM and AD-related biomarkers (e.g., inflammatory cytokines) were observed, many studies failed to clarify direct causal pathways. The gut-brain axis involves overlapping immune, metabolic, and neural pathways [188]. For example, SCFA-producing genera such as *Roseburia* are hypothesized to exert anti-inflammatory effects through modulation of NF-κB and microglial activation, but their precise molecular signaling remains unstudied [78]. This lack of mechanistic insight makes it difficult to identify the key pathways involved in AD progression.

Third, the predominance of cross-sectional human studies limits causal interference. Longitudinal designs and experimental approaches, including fecal microbiota transplantation (FMT) or colonization studies using gnotobiotic models are needed to establish causality [189, 190]. For instance, while associations between taxa such as *Escherichia/Shigella* and cytokines like IL-1β have been reported, it remains unclear whether these microbes directly drive inflammation or reflect downstream disease processes [191]. The exclusion of animal studies may have further limited mechanistic insight [192].

Finally, the search strategy was restricted to Scopus and PubMed and to English-language publications, which may have introduced selection and publication bias by excluding relevant studies published in other languages.

## Conclusions

This review highlights GM as a key modulator of AD through interactions between microbial composition and metabolic and inflammatory pathways. Dysbiosis in AD is characterized by a shift toward pro-inflammatory, LPS-producing taxa (e.g., *E. coli*) alongside depletion of beneficial SCFA-producing bacteria such as *Faecalibacterium* and *Roseburia*. These changes have been associated with increased neuroinflammation, impaired BBB integrity, and disrupted Aβ clearance, collectively contributing to disease progression.

These findings support the therapeutic potential of targeting the gut microbiome. Interventions aimed at restoring microbial balance, including probiotics, prebiotics, dietary modification, and FMT, may enhance SCFA production and reduce LPS-mediated inflammation [193–195]. However, translation into clinical practice is challenged by functional variability within taxa, complex microbial interactions, and inter-individual differences, highlighting the need for more precise, mechanism-based approaches.

Future research should prioritize longitudinal and mechanistic studies to clarify causal relationships between GM and AD, including the identification of key taxa and their interactions with host genetics and environmental factors. Integration of multi-omics approaches, such as metagenomics and metabolomics, with clinical biomarkers, neuroimaging, and immune profiling will be critical for defining mechanistic pathways and identifying clinically relevant microbial signatures [196–198]. Addressing bacterial populations and their mechanisms will deepen our understanding of how GM contributes to AD, bridging the gap between research and clinical practice.

### Clinical Implications

Emerging evidence suggests that GM may serve both as a biomarker and a therapeutic target in AD. Alterations such as reduced abundance of SCFA-producing bacteria (*Faecalibacterium*, *Roseburia*) and increased pro-inflammatory taxa (e.g., *E. coli*) have been associated with metabolic dysregulation, including reduced SCFAs and altered BA and LPS profiles, as well as key AD biomarkers such as Aβ, p-tau, and YKL-40 [48, 72].

Interventions aimed at modulating GM, including probiotics, prebiotics, dietary strategies, and FMT, show potential to improve cognitive outcomes and reduce neuroinflammation [197]. However, current evidence remains limited by small sample sizes, methodological heterogeneity, and variability in participant characteristics, including diet, medication use, and comorbidities. These differences limit the comparability of findings and make it difficult to draw firm conclusions [8, 72].

Future studies should focus on larger, longitudinal studies with biomarker-confirmed AD diagnoses and randomized controlled trials to establish causality. Integration of multi-omics techniques, including metagenomics, metatranscriptomics, and metabolomics, with clinical and neuroimaging data will be critical to determine whether microbiota alterations are causal or consequential in AD [198, 199]. Standardization of methodologies and data sharing will further improve reproducibility and facilitate the development of reliable microbiome-based diagnostics and therapies.[200, 201] 

## Data Availability

No datasets were generated or analysed during the current study.

## Abbreviations

Aβ: Amyloid-beta
AD: Alzheimer’s disease
ADAS-Cog: Alzheimer’s Disease Assessment Scale–Cognitive subscale
ADL: Activities of daily living
Anti: Anti-inflammatory
BA: Bile acid
CDR: Clinical dementia rating
CDT: Clock drawing test
CERAD: Consortium to Establish a Registry for Alzheimer’s Disease
CSF: Cerebrospinal fluid
DL-5-MTP: DL-5-Methoxytryptophol
DSM-IV: Diagnostic and Statistical Manual of Mental Disorders, fourth edition
GM: Gut microbiota
IL-1β: Interleukin-1 beta
IL-6: Interleukin-6
IL-8: Interleukin-8
IL-10: Interleukin-10
INF-γ: Interferon-gamma
IP-10: Interferon gamma–induced protein 10
IWG-2 (2014): International Working Group on the Diagnosis of Alzheimer’s Disease, 2nd Meeting (2014)
LCA: Lithocholic acid
LPS: Lipopolysaccharide
MMSE: Mini-Mental State Examination
MoCA: Montreal cognitive assessment
MRI: Magnetic resonance imaging
ND: Not determined
NIA-AA: National Institute on Aging and Alzheimer’s Association
NINCDS-ADRDA: National Institute of Neurological and Communicative Disorders and Stroke–Alzheimer’s Disease and Related Disorders Association
NINDS/ADRDA: National Institute of Neurological Disorders and Stroke/Alzheimer’s Disease and Related Disorders Association
p-gp: P-glycoprotein
PCR: Polymerase chain reaction
PET: Positron emission tomography
Pro: Pro-inflammatory
SCFA: Short-chain fatty acids
TNF-α: Tumor necrosis factor-alpha
YKL-40: Chitinase-3-like protein 1
